# Caveolin-1 Is a Critical Determinant of Autophagy, Metabolic Switching, and Oxidative Stress in Vascular Endothelium

**DOI:** 10.1371/journal.pone.0087871

**Published:** 2014-02-03

**Authors:** Takashi Shiroto, Natalia Romero, Toru Sugiyama, Juliano L. Sartoretto, Hermann Kalwa, Zhonghua Yan, Hiroaki Shimokawa, Thomas Michel

**Affiliations:** 1 Cardiovascular Division, Department of Medicine, Brigham and Women’s Hospital, Harvard Medical School, Boston, Massachusetts, United States of America; 2 Department of Cardiovascular Medicine, Tohoku University Graduate School of Medicine, Sendai, Japan; University of Miami School of Medicine, United States of America

## Abstract

Caveolin-1 is a scaffolding/regulatory protein that interacts with diverse signaling molecules. Caveolin-1^null^ mice have marked metabolic abnormalities, yet the underlying molecular mechanisms are incompletely understood. We found the redox stress plasma biomarker plasma 8-isoprostane was elevated in caveolin-1^null^ mice, and discovered that siRNA-mediated caveolin-1 knockdown in endothelial cells promoted significant increases in intracellular H_2_O_2_. Mitochondrial ROS production was increased in endothelial cells after caveolin-1 knockdown; 2-deoxy-D-glucose attenuated this increase, implicating caveolin-1 in control of glycolytic pathways. We performed unbiased metabolomic characterizations of endothelial cell lysates following caveolin-1 knockdown, and discovered strikingly increased levels (up to 30-fold) of cellular dipeptides, consistent with autophagy activation. Metabolomic analyses revealed that caveolin-1 knockdown led to a decrease in glycolytic intermediates, accompanied by an increase in fatty acids, suggesting a metabolic switch. Taken together, these results establish that caveolin-1 plays a central role in regulation of oxidative stress, metabolic switching, and autophagy in the endothelium, and may represent a critical target in cardiovascular diseases.

## Introduction

Caveolin-1 is a scaffolding/regulatory protein localized in plasmalemmal caveolae that modulates signaling proteins in diverse mammalian cells, including endothelial cells and adipocytes [1]. Plasmalemmal caveolae have a distinctive lipid composition, and serve as microdomains for the sequestration of signaling proteins including G proteins, receptors, protein kinases, phosphatases, and ion channels. In the vascular endothelium, a key caveolin-1 binding partner is the endothelial isoform of nitric oxide synthase (eNOS) [2]. eNOS-derived nitric oxide (NO) plays a central role in vasorelaxation; the binding of caveolin-1 to eNOS inhibits NO synthesis. Caveolin-1^null^ mice show enhanced NO-dependent vascular responses, consistent with the inhibitory role of caveolin-1 in eNOS activity in the vascular wall [3], [4]. Yet the phenotype of the caveolin-1^null^ mouse goes far beyond effects on cardiovascular system: caveolin-1^null^ mice have profound metabolic abnormalities [5], [6] and altered redox homeostasis, possibly reflecting a role of caveolin-1 in mitochondrial function [6], [7]. Caveolin-1^null^ mice also develop cardiomyopathy and pulmonary hypertension [8], associated with persistent eNOS activation secondary to the loss of caveolin-1. This increase in NO leads to the inhibition of cyclic GMP-dependent protein kinase due to tyrosine nitration [9].

Caveolin-1^null^ mice show increased rates of pulmonary fibrosis, cancer, and atherosclerotic cardiovascular disease [1], all of which are pathological states associated with increased oxidative stress. Functional connections between caveolin and oxidative stress have emerged in several recent studies. The association between oxidative stress and mitochondria has stimulated studies of caveolin in mitochondrial function and reactive oxygen species (ROS). The muscle-specific caveolin-3 isoform may co-localize with mitochondria [10], and mouse embryonic fibroblasts isolated from caveolin-1^null^ mice show evidence of mitochondrial dysfunction [7]. Endothelial cell mitochondria have been implicated in both physiological and pathophysiological pathways [11], and eNOS itself may synthesize ROS when the enzyme is “uncoupled” by oxidation of one of its cofactors, tetrahydrobiopterin. At the same time, the stable ROS hydrogen peroxide (H_2_O_2_) modulates physiological activation of phosphorylation pathways that influence eNOS activity [12], [13]. Clearly, the pathways connecting caveolin, eNOS, mitochondria, and ROS metabolism are complex yet critical determinants of cell function– both in normal cell signaling and in pathological states associated with oxidative stress.

Analyses of the roles of caveolin in metabolic pathways have exploited gene-targeted mouse models focusing on the metabolic consequences of caveolin-1 knockout on energy flux in classic “energetically active” tissues of fat, liver, and muscle [6]. The role of the vascular endothelium as a determinant of energy homeostasis has been recognized only more recently. For example, endothelial cell-specific “knockout” of insulin receptors [14] was found to affect systemic insulin resistance, and we found that endothelial cell-specific knockout of PPAR-gamma [15] affects organismal carbohydrate and lipid metabolism. In turn, metabolic disorders can markedly influence endothelial signaling pathways: hyperglycemia suppresses NO-dependent vascular responses [16], while high glucose treatment of cultured endothelial cells increases intracellular levels of ROS, including H_2_O_2_ [17].

The present studies have used biochemical, cell imaging, and metabolomic approaches to explore the roles of caveolin-1 in endothelial cell redox homeostasis, and have identified novel roles for caveolin-1 in modulation of endothelial cell oxidative stress, metabolic switching, and autophagy.

## Materials and Methods

### Ethics statement

Protocols for all animal experiments were approved by the Harvard Medical Area Standing Committee on Animals, which adheres strictly to national and international guidelines for animal care and experimentation.

### Materials

Anti-caveolin-1 antibody was from BD Transduction Laboratories (Lexington, KY). Antibodies against apoptosis induction factor (AIF), LC3B and cytochrome c oxidase IV were from Cell Signaling Technologies (Beverly, MA). Amplex Red, 5-(and-6)-chloromethyl-2’,7’dichlorodihydrofluorescein diacetate acetyl ester (CM-H_2_DCFDA), MitoSOX Red, MitoTracker Green FM and tetramethyl rhodamine methyl ester (TMRM), Lipofectamine 2000, Alexa Fluor 488- and Alexa Fluor 568-coupled secondary antibodies were from Invitrogen. Cyto-ID autophagy probe and VAS-2870 was from Enzo Life Science. GSH/GSSG-Glo Assay kit was from Promega Corporation (Madison, WI). 8-isoprostane Affinity Purification Kit, 8-isopronstane EIA kit and catalase assay kit were from Cayman Chemicals. Bovine aortic endothelial cells (BAEC) were obtained from Genlantis (San Diego, CA). Cell culture media was from Gibco, Life science. Fetal bovine serum was purchased from HyClone Laboratories. All other reagents were from Sigma.

### Cell culture and siRNA transfection

BAEC were maintained in Dulbecco’s modified Eaglés medium (DMEM) with glucose (5.5 mM) and supplemented with FBS (10% v/v). BAEC were cultured in gelatin-coated culture dishes, and studies were performed prior to cell confluence between passages 5 and 8. For siRNA transfection experiments, cells were transfected with the indicated siRNA duplex targeting constructs (30 nM) with Lipofectamine (0.15% v/v); medium was changed 5 h after transfection. 24–48 h later, cells were split (1∶5) and were analyzed 72 h after transfection. siRNA targeting constructs for caveolin-1, eNOS, Rac-1 and AMPKα-1 have been previously characterized in detail [18], [19]. In some “double knockdown” experiments, cells were transfected with two different siRNA targeting constructs at a total siRNA concentration of 60 nM, comprising 30 nM of each targeting siRNA or control siRNA as needed to yield a final concentration of 60 nM; for these experiments, Lipofectamine (0.15% v/v) was used.

### Amplex red assay

72 h following transfection with siRNA targeting constructs, BAEC were washed two times with Dulbecco’s phosphate-buffered saline (dPBS) and incubated with dPBS containing Amplex Red (50 µM), horseradish peroxidase (0.1 U/ml) and superoxide dismutase (10 U/ml) for 30 min in the dark. In some experiments, pharmacological inhibitors were added 1 h before starting the Amplex Red assay. Following treatments, fluorescence intensity was determined in cell supernatants at 530 nm excitation and 590 nm emission wavelengths. Following the Amplex Red assay, cells were collected for protein determinations and/or immunoblot analyses in order to normalize fluorescence values to levels of protein abundance.

### Plasma 8-isoprostane determination

8–10 week-old male caveolin-1^null^ and age-matched wild-type control mice (Jackson Labs) were starved overnight; blood was collected from the tail vein, treated with 20 µM indomethacin and centrifuged at 3000 rpm for 10 min at 4°C to separate the plasma fraction. Plasma samples were immediately treated with butylated hydroxytoluene (BHT, 50 µg/mL) and stored at -80° C until analysis. 8-isoprostanes determination was performed using a commercial kit (Cayman Chemicals) according to the manufacturer’s protocol.

### HyPer2 lentivirus cloning and tail vein injection

Recombinant lentivirus expressing the H_2_O_2_ biosensor HyPer2 (described in detail in [13]) was infused through the tail vein (250 µl of 10^8^ pfu/ml) of wild-type or caveolin-1^null^ adult male mice (8–10 wk old). Two weeks after lentivirus injection, mice were sacrificed and the descending aortae carefully dissected and fixed with 4% paraformaldehyde, and then transverse sections of aorta were mounted on slides and processed for confocal microscopy. Metamorph software was used to determine the HyPer2 ratio, as we have previously described[13]. The intracellular HyPer2 ratio was calculated for individually selected endothelial cells to determine levels of intracellular H_2_O_2_.

### Glutathione determination

siRNA-transfected endothelial cells were trypsinized 48 h following transfection, diluted 1∶8 and seeded in 96 multi-well dishes pre-coated with gelatin; parallel tissue culture dishes were prepared for protein determination and western blot analysis. 24 h later, the GSH/GSSG ratio was determined using GSH/GSSG-Glo Assay Kit (Cayman) according to manufacturer’s instructions.

### Catalase assay by aminotriazole-mediated enzyme inactivation

Cells grown in 6-well dishes were transfected with control or caveolin-1 siRNA. Forty eight hours after transfection, media was replaced by fresh cell culture media containing either 10 mM 3-amino-1,2,4-triazole (ATZ) or vehicle, and incubated for additional 21 hrs. Cells were then rinsed with PBS, scraped into 0.5 ml of potassium phosphate buffer (50 mM, pH 7) containing 1 mM EDTA and protease inhibitors cocktail. Cells were sonicated and centrifuged (13,200 rpm×15 min), and catalase activity was assayed in the supernatants using a catalase assay (Cayman Chemicals) according to the manufacturer’s instructions.

### Detection of H_2_O_2_ using Hyper2 or Hyper2-M biosensor in BAEC

BAEC cultured in DMEM without phenol red containing FBS (10%) were concurrently transfected with Hyper2 or Hyper2-M (2 µg) plasmids and with Cav-1 or control siRNAs (30 nM) using Lipofectamine (0.15%v/v). 24 h after transfection cells were split and seeded in 8 well cover slips, grown for other 24 h and then fixed using 4% PFA. Ratiometric images were acquired using 420/40 and 500/16 band-pass excitation filters and 535/30 band-pass emission filter as described [20] and analysis was performed using Metamorph software.

### Metabolomic studies

Each sample for metabolomic analysis was generated from three p100 mm dishes that were harvested, pooled, and immediately frozen in liquid nitrogen. Six independent samples for each siRNA transfection condition (control, caveolin-1 and PKA siRNA) were individually prepared for separate analysis on the GC/MS and LC/MS/MS platforms by Metabolon (Durham, NC). All cell treatments, metabolite analysis, sample preparation, quality assurance procedures, spectroscopy, and statistical analysis was performed as previously described[21]. The raw metabolomic data was normalized to the total cellular protein content (using the Bradford assay) prior to statistical analysis. The data set comprises a total 319 named biochemicals. Following log transformation and imputation with minimum observed values for each compound, Welch’s two-sample t-test was used to identify biochemicals that differed significantly between Cav-1 or PKA siRNA transfected cells and control cells. Heatmaps were generated using online software Matrix2png [22].

### Autophagy marker measurements

siRNA-transfected endothelial cells were stained and fixed 72 h after transfection using the CytoID Autophagy Detection Kit (Enzo Lifescience) according to the manufactureŕs protocol, and analyzed either by flow cytometry or by fluorescence microscopy.

## Results

### Analyses of H_2_O_2_ metabolism in cultured endothelial cells following siRNA-mediated caveolin-1 knockdown

**Figure 1:**
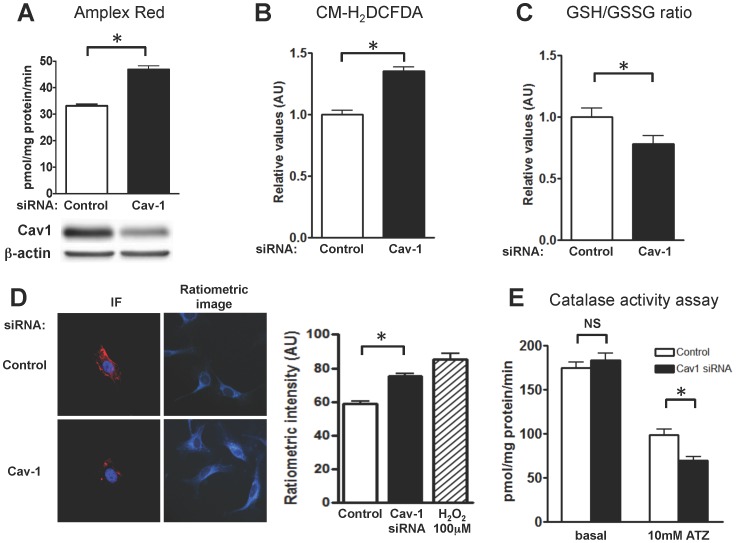
Effects of siRNA-mediated caveolin-1 knockdown on ROS levels in cultured endothelial cells. BAEC were transfected with a caveolin-1 or control siRNA, and analyzed 72 h following transfection. (A) Amplex Red assay for H_2_O_2_, pooled data from six independent experiments, each performed in duplicate. (B) Flow cytometry experiments measuring intracellular ROS levels using CM-H_2_DCFDA in control and caveolin-1 siRNA-transfected BAEC (n = 12). (C) Ratio of reduced to oxidized glutathione (GSH/GSSG ratio) in control and caveolin-1 siRNA-transfected cells (n = 8 each). (D) Intracellular H_2_O_2_ levels assessed by HyPer2 ratiometric analysis of BAEC co-transfected with caveolin-1 siRNA and Hyper2 plasmid DNA; 72 h later cells were fixed and analyzed by fluorescent microscopy; separate coverslips were analyzed for caveolin-1 immunofluorescence. *Left,* photomicrographs shows representative immunofluorescence images, with the caveolin-1 signal in red, and the DAPI nuclear stain in blue. *Right,* representative ratiometric images showing HyPer2 in control and caveolin siRNA-transfected cells; the bar graph on the far right shows pooled data from 48-50 cells, presenting quantitative analysis of the ratiometric intensity analyzed in three separate experiments. For this experiment, addition of H_2_O_2_ (100 µM) serves as a positive control for the HyPer2 biosensor. (E) Catalase activity measured in control and caveolin-1 siRNA transfected cells in the presence and absence of the inhibitor 3-amino-1,2,4-triazole (ATZ). All values are expressed as mean ± standard error of the mean (SEM). Statistical difference was assessed with unpaired t-test; * indicates p<0.05.

We explored the role of caveolin-1 in the regulation of redox metabolism in the endothelium in studies in cultured aortic endothelial cells following siRNA-mediated knockdown of caveolin-1. We utilized bovine aortic endothelial cells (BAEC), a tractable and informative cultured endothelial cell model in which caveolin-modulated pathways have been characterized [23], [24]. We used a caveolin-1 duplex siRNA targeting construct that has been validated in these cells [18], [19] and which promotes a ∼85% knockdown in caveolin-1 protein with no substantive off-target effects. As shown in Figure 1A, siRNA-mediated caveolin-1 knockdown leads to a significant increase in cellular H_2_O_2_ production, detected using the Amplex Red assay [25]. Similar results were obtained in analyses of cellular ROS levels using the fluorescent probe 5-(and-6)-chloromethyl-2′,7′-dichlorodihydrofluorescein diacetate acetyl ester (CM-H_2_DCFDA), which revealed significantly increased ROS levels following caveolin-1 knockdown (Figure 1B). We next determined the intracellular ratio of reduced to oxidized glutathione (the GSH/GSSG ratio) in caveolin-1 and control siRNA transfected cells (Figure 1C). Glutathione is a critical determinant of the intracellular redox state, and the measurement of GSH/GSSG ratio represents a robust intracellular redox indicator [26]. As shown in Figure 1C, the GSH/GSSG ratio was decreased following siRNA-mediated caveolin-1 knockdown, indicating increased endothelial cell oxidative stress following caveolin-1 knockdown. Next, we co-transfected endothelial cells with caveolin-1 or control siRNA along with a plasmid encoding the H_2_O_2_ biosensor HyPer2. We quantitated H_2_O_2_ levels by measuring the ratiometric HyPer2 fluorescence change (Figure 1D). HyPer2-transfected endothelial cells following siRNA-mediated caveolin-1 knockdown revealed a significant increase in intracellular H_2_O_2_. We measured catalase inactivation using the reagent 3-amino-2,4,5-triazole as a surrogate for H_2_O_2_ levels since the extent of aminotriazole–promoted catalase inhibition is enhanced by increases in intracellular H_2_O_2_ [17]. As shown in Figure 1E, aminotriazole-dependent catalase inactivation was markedly increased in cell lysates of endothelial cells in which caveolin-1 had been knocked down, consistent with an increase in intracellular H_2_O_2_.

### Oxidative stress in cav-1^null^ mice

**Figure 2:**
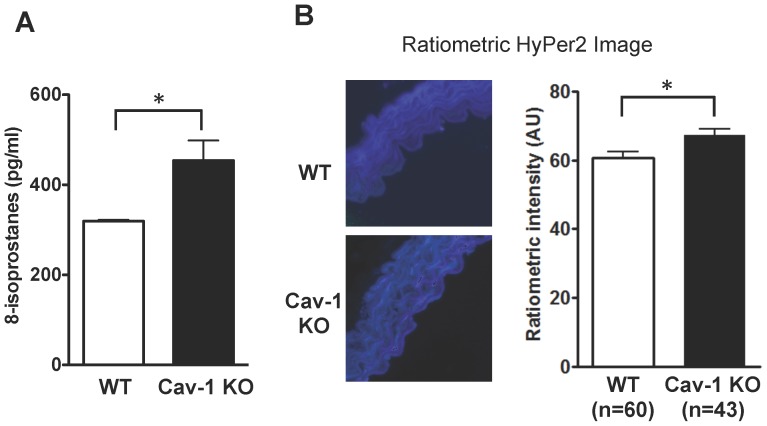
Systemic and vascular oxidative stress in caveolin-1^null^ mice. (A) 8-isoprostanes concentrations found in plasma from wild-type (wt) and caveolin-1^null^ (Cav-1 KO) mice determined in duplicate for n = 4 mice in each group; statistical difference was assessed with unpaired t-test; * indicates p<0.05 between wild-type and Cav-1^null^ mice. (B) Wild-type and Cav-1^null^ mice were infected via tail vein injection with recombinant lentivirus expressing the HyPer2 biosensor to assess H_2_O_2_ levels in vascular tissues *in situ*, then sacrificed and the descending aortae was dissected, fixed and processed for confocal microscopy to analyze the HyPer2 ratio, as we have previously described [13]. The photomicrographs on the left show representative ratiometric HyPer2 images of aortae from wild-type and Cav-1^null^ mice. The HyPer2 ratiometric signals are positive for H_2_O_2_ in both wild-type and Cav-1^null^ mouse arterial preparations, likely reflecting the prevalence of H_2_O_2_-modulated signaling pathways in the vascular wall (11,12). The bar graph on the right shows the results of quantitative analysis of HyPer2 ratiometric intensity in the vascular endothelium in these aortic preparations; the number of cells studied is shown for each. Statistical differences in endothelial cell H_2_O_2_ between wild-type and Cav-1^null^ animals were assessed with an unpaired t-test; * notes p<0.05.

To extend these findings from cultured endothelial cells to *in vivo* models, we analyzed oxidative stress in plasma and arterial preparations from cav-1^null^ mice. We measured the levels of the oxidative stress biomarker 8-isoprostanes in plasma from age- and gender-matched wild-type and cav-1^null^ mice. As shown in Figure 2A, total plasma levels of 8-isoprostanes are significantly increased in cav-1^null^ mice (454±44 vs. 319±3 pg/ml, p<0.05). In addition, we used the Hyper-2 biosensor to determine whether cellular levels of H_2_O_2_ in intact arterial preparations were altered in cav-1^null^ mice. We performed mouse tail vein injections of a recombinant lentiviral construct expressing Hyper2, which we [13] and others [27] have extensively characterized. Two weeks after infection of wild-type or cav-1^null^ mice with the HyPer2 lentivirus construct, the animals were sacrificed and mouse aortae were isolated and analyzed by fluorescence microscopy (Figure 1B). The ratiometric HyPer2 fluorescence intensity was quantitated in the vascular endothelial cells in these aortic preparations, and analyzed by a blinded observer. We established a small but statistically significant increase in HyPer2 intensity (Figure 2B), which reflects an increase in intracellular H_2_O_2_ levels in vascular endothelial cells in arterial preparations isolated from cav-1^null^ mice. Since these arterial preparations have a high background fluorescence and an unfavorable signal-to-noise ratio, we decided to pursue mechanistic studies of caveolin-1 in ROS metabolism in cultured endothelial cells.

### Intracellular sources of ROS modulated by caveolin-1

**Figure 3:**
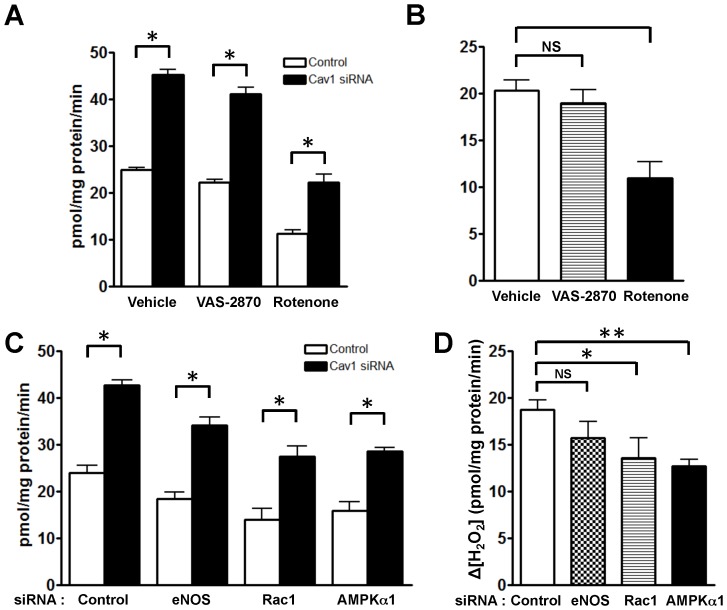
Characterization of intracellular pathways regulating caveolin-1-modulated changes in endothelial H_2_O_2_. (A), (B) Transfected BAEC were treated for 1 hour with the NADPH oxidase inhibitor VAS-2870 (10 µM) or with the mitochondrial inhibitor rotenone (10 µM), and then assayed for basal H_2_O_2_ production using Amplex Red assay (n = 6 each). (C), (D) Results of “double knockdown” experiments, cells transfected with either control or caveolin-1 siRNA were also transfected with siRNA constructs targeting eNOS, the small GTPase Rac1, or protein kinase AMPKα1. Forty-eight hours after transfection, H_2_O_2_ levels were measured by Amplex Red assay (n = 6 each). (B), (D) Absolute differences in H_2_O_2_ levels (shown as Δ[H_2_O_2_]) derived from the results shown in (A) and (C), respectively. All values are expressed as mean ± standard error of the mean (SEM). Statistical difference was assessed with unpaired t-test: * indicates p<0.05, ** indicates p<0.01.

We used small molecule inhibitors and siRNA approaches in endothelial cells to explore the caveolin-1 pathways influenced by caveolin-1 that might alter endothelial ROS. We first knocked-down eNOS [18], an enzyme that produces NO but can synthesize superoxide when the enzyme is “uncoupled” [28]. We performed Amplex Red assays but found that eNOS knockdown yielded no significant change in basal H_2_O_2_, nor in the increase in H_2_O_2_ levels seen after caveolin-1 knockdown (Figure 3C). This observation suggests that neither eNOS-derived NO nor superoxide are involved in the increase in H_2_O_2_ observed after caveolin-1 knockdown. When H_2_O_2_ levels were measured in endothelial cells pre-incubated with the eNOS inhibitor L-NAME or with the xanthine oxidase inhibitor oxypurinol following transfection with caveolin-1 siRNA, no changes in caveolin-1 modulated H_2_O_2_ levels were observed (data not shown). When siRNA-transfected endothelial cells were treated with the NADPH oxidase inhibitor VAS-2870 (Figure 3A), there was no change in the increase in H_2_O_2_ seen after caveolin-1 knockdown. However, when siRNA-transfected cells were treated with the mitochondrial inhibitor rotenone, a significant decrease in cellular H_2_O_2_ levels was observed: the increased H_2_O_2_ production usually observed following caveolin-1 knockdown was attenuated in the presence of rotenone (Figure 3A). We performed secondary plots analyzing the difference in H_2_O_2_ levels between caveolin-1 and control siRNA-transfected cells treated with these different inhibitors (Figure 3B). Rotenone attenuated the increase in H_2_O_2_ levels seen after caveolin-1 knockdown, indicating that mitochondrial electron transport is a major source of the observed increased in ROS production. We were intrigued to observe a similar inhibition of H_2_O_2_ production following siRNA-mediated knockdown of the key “energy gauge” kinase AMP-activated protein kinase (AMPK) or the regulatory small GTPase Rac-1 (Figure 3C and 3D), suggesting that the increase in ROS observed after caveolin-1 knockdown may broadly reflect metabolic dysfunction in these cells.

### siRNA-mediated caveolin-1 knockdown affects mitochondrial function

**Figure 4:**
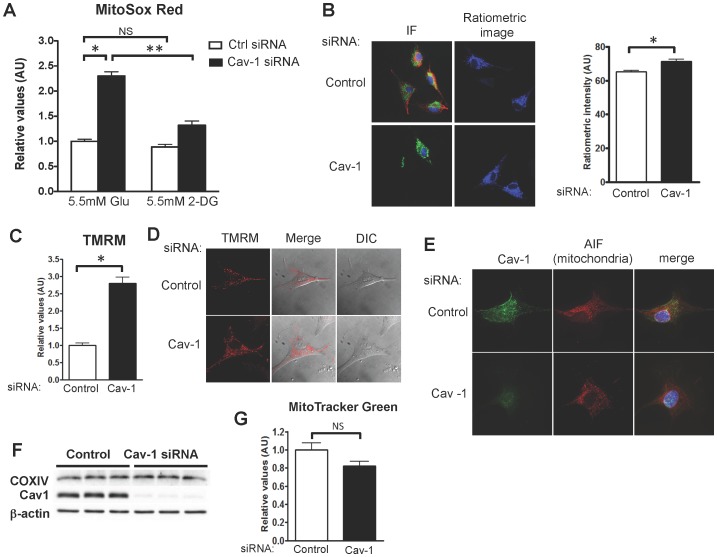
Effects of caveolin-1 knockdown on endothelial mitochondria ROS metabolism, mitochondrial function and abundance. (A) Analysis of mitochondrial ROS using the dye MitoSox Red in cells incubated in low-glucose DMEM medium (5.5 mM D-glucose) or after 6 hours in D-glucose-free DMEM medium containing 5.5 mM 2-deoxy-D-glucose (2DG). MitoSox fluorescence was analyzed by flow cytometry (n = 12). (B) BAEC were transfected with plasmids encoding mitochondria-targeted HyPer2 (HyPer-M) concomitantly with caveolin-1 or control siRNA. The cells were fixed 72 h after transfection and analyzed by HyPer2 ratiometric imaging and by immunofluorescence staining using the confocal microscope. *Left*, representative immunofluorescence, with caveolin-1 immunofluorescence signal in red and the HyPer2-M ratiometric image in green; the nuclear DAPI stain is in blue. The bar graph on the right shows the quantitative analysis of ratiometric signals pooled from analysis of 42 cells from three independent experiments. (C) and (D) Mitochondrial membrane potential assessed by labeling cells with the dye TMRM. Membrane potential was both quantitated by flow cytometry (panel C, n = 8) and analyzed by live cell imaging (D). (E) Co-localization of caveolin-1 with mitochondria in endothelial cells. Representative confocal images of endothelial cells transfected with caveolin-1 or control siRNA and subsequently stained with antibodies directed against caveolin-1 (cav-1, green) or the mitochondrial protein marker apoptosis inhibitor factor (AIF, red). Co-localization (yellow) is seen in the right-hand images, which also show nuclear staining with DAPI (blue). This experiment was repeated three times with similar results. (F) Representative immunoblots following siRNA-mediated caveolin-1 knockdown in endothelial cells. Immunoblots were probed with the mitochondrial marker protein cytochrome c oxidase subunit 4 (COX-IV), with β-actin as a loading control or with caveolin-1 to determine the extent of protein knockdown. (G) Mitochondrial abundance in control or caveolin-1 siRNA transfected cells assessed by flow cytometry using the probe MitoTracker Green FM (n = 4 each). All values are expressed as mean ± standard error of the mean (SEM). Statistical differences were assessed with unpaired t-tests: * indicates p<0.05, ** indicates p<0.01, NS, not significant.

We next explored the effects of caveolin-1 knockdown on mitochondrial ROS metabolism using the chemical probe MitoSox [29]. siRNA-mediated caveolin-1 knockdown led to a significant increase in MitoSox fluorescence compared to control siRNA-transfected cells (Figure 4A), indicating an increase in mitochondria-derived ROS. We next utilized a variant of the H_2_O_2_ biosensor Hyper2 specifically targeted to mitochondria (called HyPer2-M [27]) to analyze mitochondrial H_2_O_2_ levels following siRNA-mediated caveolin-1 knockdown. Caveolin-1 knockdown leads to a significant increase in Hyper2-M fluorescence compared to control siRNA-transfected cells (Figure 4B). We next exploited the glycolysis inhibitor 2-deoxy-D-glucose to probe the role of glycolysis. The increase in ROS observed after caveolin-1 knockdown was entirely abrogated by 2-deoxy-D-glucose, as analyzed using MitoSox (Figure 4A). Taken together, these observations implicate caveolin-1 in the modulation of mitochondrial oxidative metabolism.

To better understand the pathways leading to an increase in mitochondrial ROS production following caveolin-1 knockdown, we used the fluorescent probe tetramethyl rhodamine methyl ester (TMRM) to measure the mitochondrial membrane potential in siRNA-transfected endothelial cells. We found that caveolin-1 knockdown led to a significant increase in mitochondrial membrane potential (Figure 4C and 4D), with no change in total mitochondria content, as assayed both in flow cytometry analyses with MitoTracker green (Figure 4G) or by analyzing levels of the mitochondrial protein cytochrome oxidase (Figure 4F). While most caveolin is targeted to plasmalemmal caveolae [30], recent reports have observed caveolin in other intracellular membrane-limited structures, including mitochondria [10]. As shown in Figure 4E a small but significant fraction of the intracellular caveolin-1 staining co-localizes with the mitochondrial marker APF-1 in endothelial cells, providing suggestive evidence for the presence of caveolin-1 in mitochondria, as previously proposed [10], [31].

### Metabolomic analysis of caveolin-1 siRNA transfected cells: evidence for autophagy

**Figure 5:**
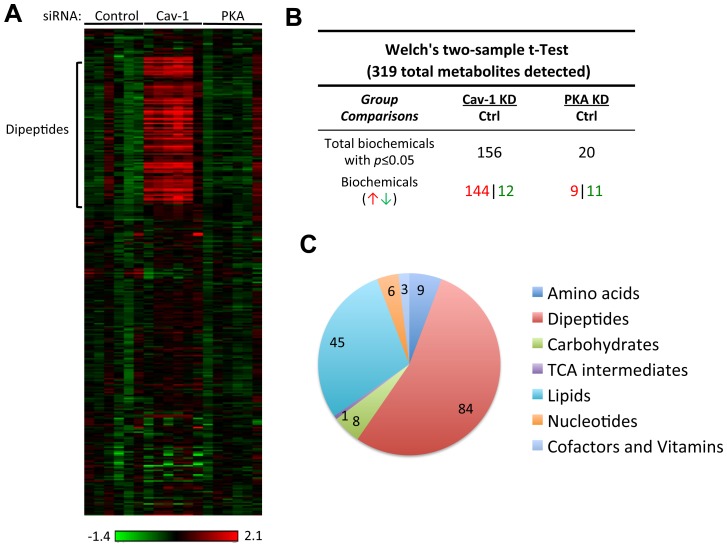
Metabolomic profile of endothelial cell lysates following caveolin-1 knockdown. Lysates prepared from endothelial cells transfected with control, caveolin-1 (Cav-1) or protein kinase A (PKA) siRNA underwent metabolic profiling as described in the text. (A) “Heat map” portraying the relative abundance of the 319 metabolites detected in cell lysates analyzed 72 h. after transfection with siRNA duplexes as shown; six independent samples were analyzed for each condition. The intensity of red or green represents the relative abundance of each specific metabolite normalized to the median value for all the samples; the corresponding logarithmic scale is shown below the graphic. Metabolites are grouped in the heat map according to the metabolic sub-pathways in which they participate, and then structural categories are ordered alphabetically within each classification. Highlighted on the left of the heat map is the region showing the most striking change following caveolin-1 knockdown, corresponding to specific dipeptides (“Dipeptides”). (B) Table with the number of different metabolites that showed statistically different abundance following caveolin-1 knockdown. (C) General distribution of the categories of metabolites that are altered in lysates prepared from cells after siRNA-mediated caveolin-1 knockdown demonstrating that marked increases in the abundance of cellular dipeptides represent the most striking alteration in the metabolic profile seen after caveolin-1 knockdown.

**Figure 6:**
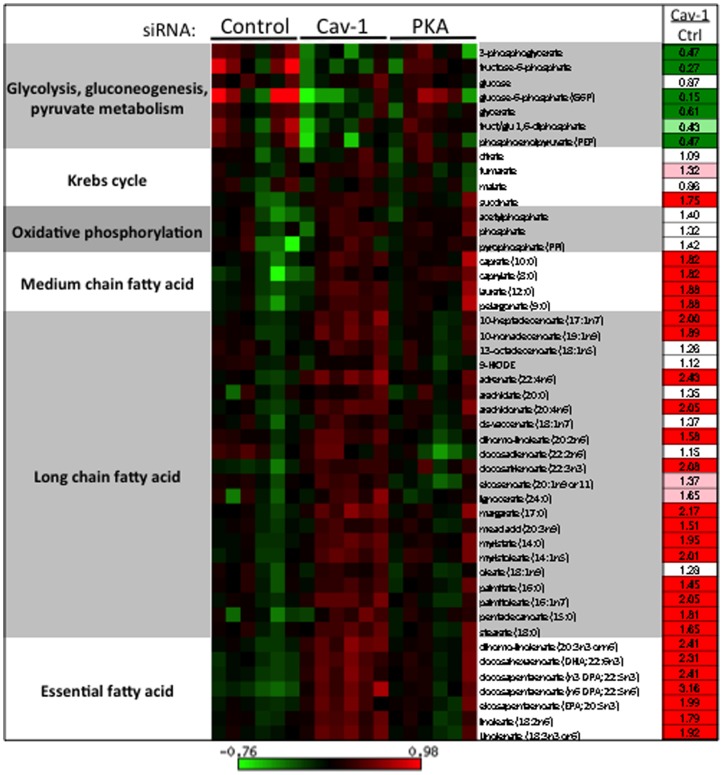
Heatmap of the relative abundance of glycolytic metabolites and free fatty acids obtained from lysates prepared from endothelial cells transfected with control siRNA or with siRNA constructs targeting caveolin-1 (Cav-1) or protein kinase A (PKA). The intensity of red or green represents the relative abundance of each specific metabolite normalized to the median value for all the samples; the corresponding logarithmic scale is shown below the graphic. The values in the column of the right side of the panel represent the fold-change of the average concentration of each metabolite of Cav-1 knockdown samples compared to control samples. For each metabolite, the media value between both groups was compared using Welch’s unpaired t-test. Bright green or red represents a significant difference between both conditions (p<0.05).

We performed unbiased metabolic profiling of endothelial cell lysates following transfection with caveolin-1 or control siRNA; we also analyzed the effects of knockdown of the cyclic AMP-dependent protein kinase (PKA). Metabolites in cell lysates obtained from six replicates per condition were analyzed by coupled LC-MS and GC-MS methods, as described [32]. After normalization to protein concentration, log transformation and imputation with minimum observed values for each compound, Welch’s two-sample *t*-tests were used to identify biochemicals that differed significantly between experimental groups. A total of 319 metabolites were identified in cell samples including amino acids, peptides, carbohydrates, lipids, nucleotides, cofactors and vitamins (Supplemental Table S1). From the identified compounds, 156 metabolites exhibited significant differences between control and caveolin-1 siRNA-transfected cells. In contrast, in cell lysates prepared from cells transfected with PKA siRNA, only 20 compounds showed significant differences compared with control siRNA-transfected cells (Fig. 5B). Figure 5A shows the hierarchical “heat map” representing the fold change obtained in the concentration of the different compounds grouped according to their metabolic pathways. The most striking difference is the remarkable increase in cellular dipeptides, seen across a broad range of dipeptide species. As shown in Figure 5C and Supplemental Table S2, among the 97 different dipeptides detected in the cell lysates, the levels of 84 discrete dipeptides are significantly increased following siRNA-mediated caveolin-1 knockdown. In addition, there is a marked decrease in the levels of glycolytic intermediates following caveolin-1 knockdown, suggesting that increased flux through glycolysis results in the depletion of glycolytic intermediates in these cells (Figure 6). Conversely, there is a significant increase in the abundance of free fatty acids in lysates from endothelial cells after caveolin-1 knockdown, suggesting either increased lipolysis and/or suppression of fatty acid oxidation. We also observed an increase in the concentration of the amino acids L-arginine, L-citrulline, and asymmetric dimethyl arginine (Supplemental Table S1); these amino acids are implicated in NOS metabolism.

**Figure 7:**
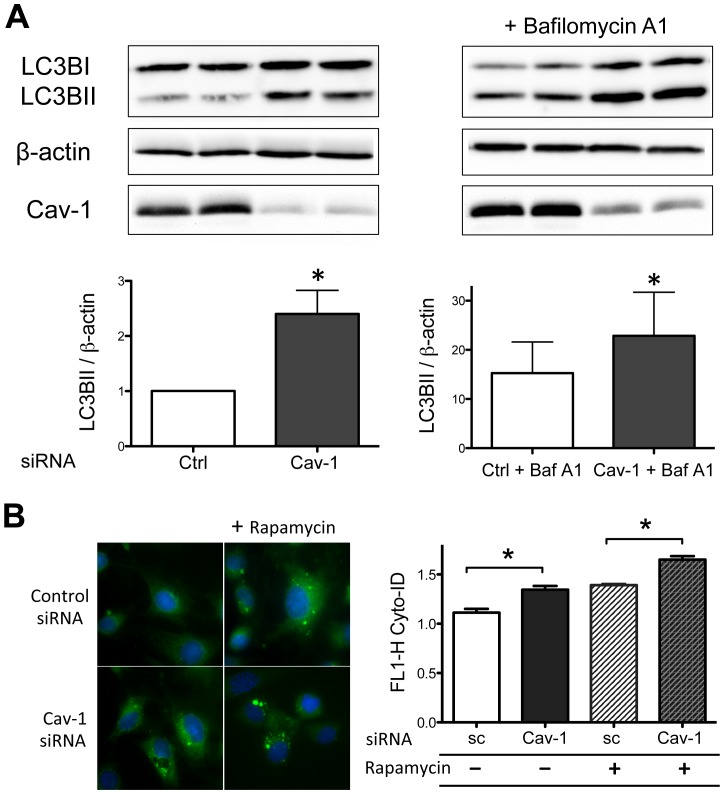
Increased autophagy in endothelial cells following siRNA-mediated caveolin-1 knockdown. (A) BAEC were transfected with caveolin-1 or control siRNA, some conditions treated with bafilomycin A1 (20 nM, 4 h), and then cell lysates were resolved by SDS-PAGE and analyzed by immunoblot using LC3B antibody; β-actin was used as loading control. (B) BAEC were transfected with caveolin-1 or control siRNA and then stained with the autophagy marker dye Cyto-ID Green 72 h following transfection. As a positive control for autophagy, some samples were treated with rapamycin (10 nM) for 18 h before labeling with Cyto-ID Green. *Left panel* shows representative microscopic images of autophagic vacuoles in endothelial cells transfected and treated as shown. *Right panel* shows the results of pooled data derived from the quantification of autophagic vacuoles by flow cytometry. All values are expressed as mean ± standard error of the mean (SEM). Statistical difference was assessed with unpaired t-test; * indicates p<0.05.

We hypothesized that the increase in endothelial cell dipeptides seen after caveolin-1 knockdown represents an increase in autophagy [33]. We used two complementary methods for the quantitation of autophagy in endothelial cells. siRNA-mediated caveolin-1 knockdown led to a striking increase of the autophagic marker protein LC3B-II, indicating an increase in cellular autophagy (Figure 7A). Treatment of control or caveolin-1 siRNA-transfected cells with the lysosomal inhibitor bafilomycin A1 (20 nM for 4 h) markedly increased LC3B-II (Figure 7A), indicating that the increase in LC3B-II seen after caveolin-1 knockdown represents an increase in autophagy flux and not a blockade in the terminal stages of the autophagy pathway [34]. As shown in Figure 7B, siRNA-mediated caveolin-1 knockdown led to a striking increase in the signal of the autophagic marker CytoID-Green, quantitated either by fluorescence microscopy (Figure 7B, left panel) or by flow cytometry (Figure 7B, right panel).

## Discussion

Many cardiovascular disease states are associated with increased levels of reactive oxygen species. However, clinical trials exploring the efficacy of antioxidant therapies for the treatment of vascular disease have failed to show any benefit. Reactive oxygen species arise from diverse intracellular and extracellular sources in the vascular wall, and there appears to be a fine balance between the physiological roles of the stable ROS hydrogen peroxide–observed at low H_2_O_2_ concentrations– and the pathological effects of higher ROS concentrations [35], [36]. The effectiveness of future antioxidant therapies for vascular disease may be influenced by the development of a deeper understanding of the intracellular pathways that modify ROS metabolism in the endothelium. The present studies provide new information linking the key scaffolding/regulatory protein caveolin-1 to oxidative stress and autophagy in vascular endothelial cells.

These studies have used a combination of cell imaging, biochemical, and metabolomic approaches to explore the roles of caveolin-1 in oxidative metabolism in the endothelium. Given the complex metabolic phenotype found in caveolin-1^null^ mice [3], [4], [6], we were intrigued to discover (Figure 2A) that caveolin-1^null^ mice have increased plasma levels of 8-isoprostanes, a reliable plasma biomarker of oxidative stress [37]. Imaging studies performed in aortic preparations isolated from caveolin-1^null^ mice expressing the H_2_O_2_ biosensor Hyper2 demonstrated increased endothelial H_2_O_2_ compared with wild-type mice (Figure 2B), consistent with the increase in systemic oxidative stress seen in plasma from the caveolin-1^null^ mouse.

In order to probe the mechanisms whereby caveolin-1 modulates endothelial metabolism, we used RNA interference methods to explore oxidative pathways in cultured endothelial cells. We used several independent methods to quantitate ROS in these cells in order to avoid potential artifacts that might confound any single ROS assay. Studies of ROS levels performed using both the Amplex Red assay (Figure 1A) as well as measurements using the redox-sensitive fluorophore CM-H_2_DCFDA (Figure 1B) demonstrated marked increases in endothelial H_2_O_2_ in caveolin-1 siRNA-transfected cells compared to control siRNA-transfected cells. We used two other independent assays for measuring H_2_O_2_ intracellular levels: the Hyper2 biosensor in cell imaging and assays of aminotriazole-dependent catalase inhibition [38]. Both methods verified the presence of increased intracellular levels of H_2_O_2_ after caveolin-1 knockdown (Figure 1D and 1E). We found that the ratio of reduced to oxidized glutathione (the GSH/GSSG ratio) was significantly decreased following caveolin-1 knockdown (Figure 1C), again consistent with an increase in oxidative stress caused by caveolin-1 knockdown. These independent ROS assays lead us to a robust conclusion that siRNA-mediated caveolin-1 knockdown promotes a significant increase in endothelial cell oxidative stress.

We next used a series of siRNA targeting constructs as well as pharmacological inhibitors to identify the intracellular source(s) of the increased ROS levels observed following caveolin-1 knockdown. Enzymatic sources for oxidative stress in endothelial cells include members of the NADPH oxidase family [39], xanthine oxidases [40] and “uncoupled” eNOS [28]. However, neither the NADPH oxidase inhibitor VAS-2870, nor the xanthine oxidase inhibitor oxypurinol, nor the NOS inhibitor L-NAME blocked the increase in ROS seen after caveolin-1 knockdown. siRNA-mediated knockdown of the AMP-activated protein kinase (AMPK) partially attenuated the increase in ROS seen after caveolin-1 knockdown, providing suggestive evidence that caveolin-1 may modulate endothelial energy metabolism as part of its effect on autophagy and ROS abundance. Similarly, after siRNA-mediated Rac1 knockdown, the increase in ROS seen after caveolin-1 knockdown is partially attenuated. We speculate that this reflects the role of Rac1 in the modulation of signaling pathways controlled by caveolin-1 (18, 19). The quantitatively most important source for ROS in these cells is from mitochondrial electron leakage during oxidative metabolism. We found the mitochondrial inhibitor rotenone significantly attenuated the increase in ROS observed after caveolin-1 knockdown, suggesting that mitochondrial dysfunction is responsible for the increase in H_2_O_2_ levels observed after caveolin-1 knockdown. This interpretation was confirmed using the mitochondrial ROS sensor MitoSox Red [29] (Figure 4A) as well as using the mitochondria-targeted H_2_O_2_ biosensor HyPer2-M (Figure 4B).

Caveolin-1 is clearly an important regulator of eNOS in endothelial cells: the absence of caveolin-1 leads to increased activity of the enzyme, with a concomitant increase in NO production [41]. Since NO is a competitive inhibitor of oxygen in the cytochrome oxidase present in mitochondrial complex IV, increased NO production could promote mitochondria inhibition by attenuating the terminal phase of the mitochondrial electron transport chain complex, leading to electron leakage and superoxide formation. In addition, upregulation of eNOS activity could also promote depletion of arginine substrate and enzyme “uncoupling” to eNOS-dependent generation of O_2_^.-^. However, neither siRNA-mediated eNOS knockdown nor pharmacological inhibition of eNOS blocked the effect of caveolin-1 knock down on increased ROS production in endothelial cells, suggesting instead that caveolin-1 has a direct effect on mitochondrial oxidative metabolism. We found that siRNA-mediated caveolin-1 knockdown promoted a striking increase in the mitochondrial production of oxidant species, associated with mitochondrial membrane hyperpolarization. In addition, we found that caveolin-1 knockdown led to a metabolic switch in cellular energy utilization, with preferential utilization of the glycolytic pathway over free fatty acid consumption, concomitant with activation of the autophagy pathway. An important caveat in our conclusions on the role of caveolin-1 in metabolic switching is that these studies were conducted in cells cultured only with a carbohydrate source, without analyzing differential effects of lipids; metabolic flux in the presence of different energy sources was not directly measured. The role of caveolin-1 in mitochondrial function and redox modulation has not previously reported in endothelial cells, nor have prior studies connected these responses with cellular autophagy. However, recent reports have suggested that caveolin-1 may modulate mitochondrial function in diverse cell types [6], [7], [10]. Indeed, we could detect co-localization between caveolin-1 and the mitochondrial marker APF-1 in control endothelial cells using confocal microscopy (Figure 4E), perhaps reflecting a direct effect of caveolin-1 on mitochondrial membrane function.

In addition to the modulation of mitochondrial function by caveolin-1, our data also implicate glycolysis as an important source of the substrates and reducing equivalents provided to the mitochondrial electronic transport chain in these cells. We found (Figure 4A) that the glycolytic inhibitor 2-deoxyglucose effectively abrogated the increase in ROS seen following caveolin-1 knockdown. Results of our metabolomic analysis (Figure 6) provide another clue to a central role for glycolysis. The fact that five intermediates of the glycolytic pathway are present at a significant lower level in lysates from caveolin-1 siRNA transfected endothelial cells- accompanied by a striking increase in free fatty acids following caveolin-1 knockdown- strongly suggests that glucose is being preferentially utilized as an energetic substrate when caveolin-1 abundance is suppressed. Previous studies of caveolin-1 cancer-associated fibroblasts also revealed a metabolic switch from an oxidative mitochondrial phenotype to a glycolytic phenotype [1]. Possibly this increased reliance on glycolysis over fatty acid oxidation reflects a compensatory mechanism that avoids the generation of excessive mitochondria-derived ROS that could have deleterious effects.

Perhaps the most dramatic and surprising cellular response observed following siRNA-mediated caveolin-1 knockdown was the activation of the autophagy pathway, which we quantitated using two independent assays for autophagy (Figure 7). The dramatic increase in the level of cytosolic dipeptides following caveolin-1 knockdown (Figure 5A) is consistent with activation of autophagy [42]. Autophagy is a regulated catabolic pathway that promotes lysosome-mediated degradation of proteins and organelles inside a cell. Autophagy was originally described as a “self-eating” pathway activated by cells under starvation conditions or in the face of energy deprivation, with the consequence that starving or stressed cells could generate intracellular molecules that are essential for cell homeostasis. However, is now widely accepted that even under normal nutritional states, autophagy has a role in normal cellular homeostasis by mediating the turnover of long-lived proteins. We found that caveolin-1 knockdown in endothelial cells led to a marked increase in intracellular dipeptide levels across a very large number (∼85) of unrelated dipeptides that have no discernable common structural characteristics (Supplemental Table S2). The striking increase in intracellular dipeptides observed in endothelial cell lysates after siRNA-mediated caveolin-1 knockdown probably arises from the general breakdown of intracellular proteins by lysosomal proteases, and we speculate that these dipeptides are probably the products of general proteolytic degradation and are not generated by specific dipeptidyl-peptidases.

Oxidative stress may play a role in autophagy, although the molecular mechanisms remain incompletely understood [33], [43], [44], [45]. Activation of autophagy following caveolin-1 knockdown may reflect an adaptive response to the marked increase in oxidative stress caused by the decrease in caveolin-1 protein expression in endothelial cells. Modulation of endothelial cell autophagy may represent another critical control point in the modulation of vascular function in disease states associated with oxidative stress.

## Supporting Information

Table S1 **Metabolite profile of endothelial cells 72 h after transfection with control, caveolin-1, or PKA siRNA.** Data Set 1 shows the raw values and Data Set 2 shows the normalized and imputed values for metabolites analyzed following siRNA-mediated knockdown using control, caveolin-1, or PKA siRNA in endothelial cells. These data provide the basis for the heat maps shown in Figure 5 and 6.
(XLSX)

Table S2 **Dipeptides detected in endothelial cells after transfection with control or caveolin-1 siRNA.** This table shows the heat map associated with the statistical analysis of quantitative metabolomic data for dipeptides following siRNA-mediated caveolin-1 knockdown in endothelial cells.
(XLSX)
